# Changes in nutritional management after gastrointestinal cancer surgery over a 12-year period: a cohort study using a nationwide medical claims database

**DOI:** 10.1186/s40795-025-01006-4

**Published:** 2025-01-22

**Authors:** Yoshikuni Kawaguchi, Kenta Murotani, Nahoki Hayashi, Satoru Kamoshita

**Affiliations:** 1 Hepato-Biliary-Pancreatic Surgery Division, Department of Surgery, Graduate School of Medicine, The University of Tokyo, 7-3-1 Hongo, Bunkyo-Ku, Tokyo, Japan; 2 School of Medical Technology, Kurume University, 777-1 Higashikushihara-Machi, Kurume, Fukuoka Japan; 3 Biostatistics Center, Kurume University, 67 Asahi-Machi, Kurume, Fukuoka Japan; 4 Medical Affairs Department, Research and Development Center, Otsuka Pharmaceutical Factory, Inc., 2-9 Kandatsukasa-Machi, Chiyoda-Ku, Tokyo, Japan

**Keywords:** Gastrointestinal cancer surgery, Postoperative nutritional management, Early oral intake, Fasting, Real-world data

## Abstract

**Background:**

Nutritional management in patients after gastrointestinal cancer surgery has changed throughout the 2000s. However, its evolution has not been formally studied. This study aimed to evaluate changes in nutritional management using real-world data.

**Methods:**

Patient data from 2011 to 2022 were extracted from a nationwide medical claims database. Patients were divided into four groups based on their year of hospital admission: period I, 2011–2013; II, 2014–2016; III, 2017–2019; IV, 2020–2022. For each period, feeding routes in all patients and prescribed doses of parenteral energy and amino acids in fasting patients during postoperative days (POD) 1–7 were determined. The results of the four different periods were compared using statistical trend tests.

**Results:**

The study cohort was comprised of 365,125 patients. During POD 1–3, the proportion of patients administered any oral intake increased over time (I, 40.3%; II, 47.1%; III, 49.4%; IV, 54.2%; P < 0.001), while that of patients receiving parenteral nutrition (PN) decreased (I, 60.1%; II, 55.0%; III, 50.3%; IV, 45.5%; P < 0.001). Of 19,661 patients with PN alone (i.e., neither oral intake nor enteral nutrition) during POD 1–7, the median (interquartile range) prescribed doses on POD 7 of energy (kcal/kg) [I, 15.3 (10.3–21.9); II, 13.9 (8.4–20.0); III, 13.2 (7.7–19.2); IV, 12.9 (7.0–18.7); P < 0.001] and amino acids (g/kg) [I, 0.65 (0.30–0.94); II, 0.58 (0.24–0.89); III, 0.56 (0.00–0.86); IV, 0.56 (0.00–0.87); P < 0.001] both decreased over time.

**Conclusion:**

From 2011 to 2022, more patients who underwent gastrointestinal cancer surgery in Japan were administered early oral intake, while fewer patients were administered early PN. Overall, the energy and amino acid doses prescribed in PN were far below the guideline recommendations.

**Supplementary Information:**

The online version contains supplementary material available at 10.1186/s40795-025-01006-4.

## Background

Patients with gastrointestinal (GI) cancers are likely to develop disorders associated with malnutrition as a result of anorexia, digestive malabsorption, and GI obstruction, as well as metabolic disorders associated with disease treatment and progression [1]. These problems may increase the rate of complications, delay oncological adjuvant treatment, and worsen prognoses [2]. The risk of malnutrition is particularly high after GI cancer surgery because GI function is impaired and food intake is insufficient [3, 4]. Active nutritional management in these patients after GI cancer surgery is necessary to maintain adequate nutritional status, prevent malnutrition, and enhance recovery after surgery.

Enhanced recovery after surgery (ERAS) programs are increasingly used in patients who have undergone major operative procedures. The key of ERAS is early oral intake after surgery [5]. Early oral intake or enteral nutrition (EN) are also recommended in the nutritional guidelines of various countries. The recommended energy intake is 20–30 kcal/kg/day, while the recommended protein intake is 0.8–1.5 g/kg/day [6–8]. Supplemental parenteral nutrition (SPN) is recommended in patients with energetic and protein deficiencies after initiating oral intake and/or EN [7]. A recent study reported on the clinical benefits of achieving nutritional goals using SPN [9]. Thanks to advances in surgical approaches, minimally-invasive surgeries, including endoscopic, laparoscopic, and robotic procedures, have become increasingly common in the past decade [10]. Early oral intake appears to improve clinical outcomes in patients undergoing minimally-invasive surgery [11]. The dissemination of ERAS and popularization of minimally-invasive surgery have played a role in developing an environment in which medical staff can actively aim to achieve nutritional goals using early postoperative oral intake with/without EN and parenteral nutrition (PN). Long-term fasting after surgery had been the common practice until early 2000s. However, since 2005, early oral intake has been recommended by ERAS [12, 13]. Despite this, it remains unclear to what degree early oral intake has been incorporated into clinical practice.

In recent years, the use of ERAS programs and minimally-invasive surgery have expanded throughout Japan [14, 15]. However, although nutritional management in the early postoperative period after GI cancer surgery is likely to have changed over this time, no large-scale nationwide study has been reported. This study addresses this gap by evaluating changes in postoperative nutritional management in patients undergoing GI cancer surgery using a large-scale medical claims database over a period of 12 years in Japan. By conducting a nationwide analysis, this study provides an initial characterization of changes in postoperative nutritional management in Japan and provides insights into the evidence-based nutritional management post-GI cancer surgery more generally.

## Methods

### Ethical statements

This study was conducted after preparing the research protocol, obtaining an approval by the Shiba Palace Clinic Ethics Review Committee (approval no. 153969_rn-36564) and a registration to the University Hospital Medical Information Network (UMIN) Clinical Trial Registry (registration no. UMIN000053645; registration date, February 19, 2024). An opt-out option was not implemented because personal patient and hospital information obtained from the database were anonymized and there was no correspondence table. This study complied with the applicable guidelines outlined in the Strengthening the Reporting of Observational Studies in Epidemiology statement [16] and in the REporting of studies Conducted using Observational Routinely-collected health Data statement [17].

### Data source

Data was obtained from a nationwide medical claims database provided by Medical Data Vision Co., Ltd. (Tokyo, Japan), comprising approximately 42 million patients in 475 Japanese hospitals, representing approximately 27% of the acute care hospitals operating in Japan as of December 2022. The database contained information including dates of hospital admission and discharge, patient characteristics at hospital admission, medical treatments during hospitalization, medical costs during hospitalization, and clinical outcomes at the time of discharge. Diagnoses were identified using the International Classification of Diseases 10th Revision (ICD-10) codes. Medical treatments were classified using the Japan-specific codes that appeared in medical claims. Personal patient and hospital information were anonymized and there was no correspondence table.

### Patients

The cohort was comprised of patients aged 18 years or older who underwent GI cancer surgery under general anesthesia between January 2011 and December 2022. Patients whose surgical site identified from its surgical name was consistent with that identified from the cancer name were extracted using the medical claims codes shown in Additional file 1. Patient height and body weight were used to calculate ideal body weight and body mass index (BMI). Thus, patients whose height or body weight data were missing or suspected to be the result of an input error (i.e., height < 100 cm or ≥ 200 cm, body weight < 10 kg or ≥ 200 kg) were excluded from the study. Extreme values for the exclusion criteria were determined referring the previous studies [18, 19]. The study involved nutritional management during 7 days after surgery. Thus, patients who died or were discharged within 7 days after surgery were also excluded. The study cohort was divided into four groups based on their year of hospital admission: Period I, 2011–2013; II, 2014–2016; III, 2017–2019; IV, 2020–2022.

### Extracted data

Patient characteristics were selected and extracted when the variables affected the nutritional management and were clinically important.

#### Patient characteristics

Patient characteristics at the time of hospital admission extracted from the database included age, sex, height, body weight, beds in admission hospital (< 200, 200–499, or ≥ 500), admission type (emergency or elective), ICD-10 disease diagnosis, activities of daily living (using the Barthel Index [20]), smoking history, and Tumor-Node-Metastasis (TNM) cancer stage [21]. Variables used in the study included age (categorized as < 60, 60–69, 70–79, 80–89, or ≥ 90 years), BMI (calculated using height and body weight; categorized based on the World Health Organization (WHO) classification [22] as < 16, ≥ 16 to < 18.5, ≥ 18.5 to < 22.5, ≥ 22.5 to < 25, ≥ 25 to < 30, or ≥ 30), and Charlson Comorbidity Index [23, 24] (used to predict mortality based on comorbidities, calculated using ICD-10 disease diagnosis, and categorized as 0–1, 2–3, 4–5, or ≥ 6). The Barthel Index (used to assess activities of daily living) was expressed as the range from 0 (requiring full assistance) to 100 (requiring no assistance) and categorized as 0, 5–40, 45–60, 65–95, or 100. Malnutrition was defined as a BMI < 18.5 in patients aged < 70 years and a BMI < 20 in patients aged ≥ 70 years (referring to low BMI in the Asian Global Leadership Initiative on Malnutrition criteria [25]). The level of food intake independence was categorized as requiring either no assistance, partial assistance, or full assistance, and was based on the Barthel Index Feeding score.

#### Preoperative medical treatments

Preoperative data extracted from the database was based on the medical claims codes shown in Additional file 1, including oral management (i.e., support of oral intake, including swallowing and chewing; data available for 2014 and later) received [26] and the type of artificial nutrition (i.e., EN [via tube feeding] and/or PN [via intravenous solutions containing amino acids and lipid]) prescribed, from the day of hospital admission to the day before surgery. This also included types of non-surgical cancer treatment (i.e., chemotherapy and/or radiation therapy) received, from 60 days to the day prior to surgery.

#### Medical treatments and medication on day of surgery

Data on the day of surgery extracted from the database were based on the medical claims codes shown in Additional file 1. This data included information on the surgical site, the surgical method employed (laparoscopic or non-laparoscopic surgery), intravenous fluids (crystalloid, colloid, and/or albumin), transfusions (red blood cells, platelets, and/or fresh frozen plasma), and intensive care unit (ICU) admission. The surgical site was based on medical claims codes and categorized as follows: esophagus, stomach, colon, rectum, liver, gallbladder/bile duct, pancreas, or multiple organs (multiple organ surgery performed on a single day). Crystalloid fluid was categorized based on prescribed volumes as ≤ 5,000 mL, > 5,000– ≤ 10,000 mL, or > 10,000 mL. Colloid fluid, albumin, and transfusions were categorized based on prescribed volumes as 0 mL, > 0– ≤ 500 mL, or > 500 mL.

### Evaluation of changes in nutritional management

#### Postoperative feeding route

Postoperative day (POD) 1 was defined as the next day of the surgery day. Data concerning the feeding routes on POD 1, 3, 5, and 7 was extracted from the database, based on the data of medical treatments and prescribed medicines which was based on medical claims codes shown in Additional file 1. Data was expressed as either oral intake (defined as meal served), EN, PN or a combination of these. Data not fitting those categories (e.g., fluids containing carbohydrate and/or electrolytes prescribed alone) were expressed as “Other”. The proportion of patients receiving each feeding route on POD 1, 3, 5, and 7 was calculated for each group and those results were compared between the groups. In addition, the proportion of patients receiving each feeding route was also calculated for each group during the periods of POD 1 to 3, POD 1 to 5, and POD 1 to 7, and those results were compared between the groups.

#### Postoperative prescribed PN doses and nutritional goal fulfilment

For patients who were fasting from POD 1 to 7 and received PN alone, the prescribed doses, based on ideal body weight (= 22 × height [m]^2^), of parenteral energy (kcal/kg), amino acids (g/kg), and lipid (g/kg) on POD 1, 3, 5, and 7 were calculated for each group. These results were compared between the groups. The composition, prescribed doses of the parenteral nutrition products, and the ideal body weight of each patient were identified in the medical claims database and used. Propofol, an anesthetic agent containing lipid emulsion as a solvent, was included in the energy and lipid dose calculations. In addition, the proportion of patients prescribed guideline-recommended target parenteral energy (≥ 20 kcal/kg) and amino acid (≥ 0.8 g/kg) doses [6] on POD 7 was calculated for each BMI category within each group, and those results were compared between the groups.

### Statistical analysis

Continuous variables were described using medians and interquartile ranges [first quartile (Q1), third quartile (Q3)] or the mean and standard deviation (SD). Categorical variables were reported using frequencies and proportion. Missing data was treated without replacement and designated as not available. The Jonckheere-Terpstra test (for continuous variables) or the Cochran-Armitage test (for binary variables) were employed to assess trends. A two-sided test was utilized with a significance level of 5%. All statistical analyses were performed using SAS, version 9.4 (SAS Institute, Inc.; Cary, NC, USA). An independent third party, A2 Healthcare Corporation (Tokyo, Japan), conducted data management and statistical analysis.

## Results

### Patient and surgery-related characteristics

**Fig. 1:**
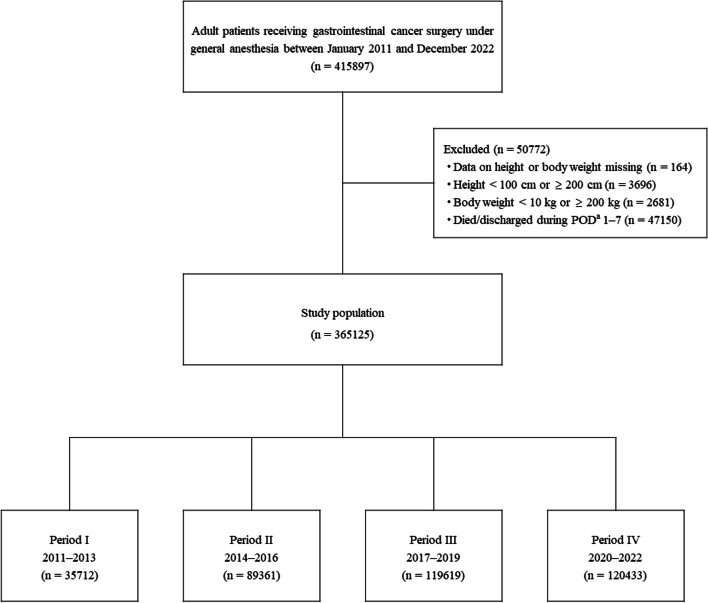
Study flow chart and disposition of patients who underwent gastrointestinal cancer surgery. ^a^Postoperative day (POD) 1 is defined as the next day of the surgery day

Of 415,897 patients who were aged 18 years or older and underwent GI cancer surgery under general anesthesia from 2011 to 2022, 365,125 patients were included in the study (Fig. 1). Among these, 218,693 (59.9%) were 70 years old or older, 135,844 (37.2%) were female, 44,211 (12.1%) had a BMI < 18.5, 71,374 (19.5%) were malnourished, and 343,025 (93.9%) required no assistance with food intake at the time of hospital admission. Between the day of admission and the day before surgery, 103,231 (28.3%) patients received oral management (Table 1).

**Table 1:** Demographic and clinical characteristics of patients who underwent gastrointestinal cancer surgery in the four time periods evaluated^a^

Characteristics	Categories	Total	Period I 2011–2013	Period II 2014–2016	Period III 2017–2019	Period IV 2020–2022
		N = 365,125	N = 35,712	N = 89,361	N = 119,619	N = 120,433
		n (%)	n (%)	n (%)	n (%)	n (%)
**Age**, *years*	18–59	50,951 (14.0)	5914 (16.6)	12,966 (14.5)	15,964 (13.3)	16,107 (13.4)
	60–69	95,481 (26.2)	10,916 (30.6)	26,482 (29.6)	31,807 (26.6)	26,276 (21.8)
	70–79	138,844 (38.0)	12,409 (34.7)	32,086 (35.9)	45,299 (37.9)	49,050 (40.7)
	80–89	73,296 (20.1)	6048 (16.9)	16,519 (18.5)	24,326 (20.3)	26,403 (21.9)
	≥ 90	6553 (1.8)	425 (1.2)	1308 (1.5)	2223 (1.9)	2597 (2.2)
**Sex**	Male	229,281 (62.8)	22,639 (63.4)	56,306 (63.0)	75,488 (63.1)	74,848 (62.1)
	Female	135,844 (37.2)	13,073 (36.6)	33,055 (37.0)	44,131 (36.9)	45,585 (37.9)
**BMI**	< 16	7842 (2.1)	748 (2.1)	1863 (2.1)	2564 (2.1)	2667 (2.2)
	≥ 16, < 18.5	36,369 (10.0)	3647 (10.2)	8882 (9.9)	11,714 (9.8)	12,126 (10.1)
	≥ 18.5, < 22.5	145,649 (39.9)	14,786 (41.4)	36,221 (40.5)	47,295 (39.5)	47,347 (39.3)
	≥ 22.5, < 25	93,240 (25.5)	9301 (26.0)	22,937 (25.7)	30,776 (25.7)	30,226 (25.1)
	≥ 25, < 30	70,679 (19.4)	6318 (17.7)	16,962 (19.0)	23,540 (19.7)	23,859 (19.8)
	≥ 30	11,346 (3.1)	912 (2.6)	2496 (2.8)	3730 (3.1)	4208 (3.5)
**Beds in admission hospital**	< 200	19,969 (5.5)	2363 (6.6)	5339 (6.0)	6357 (5.3)	5910 (4.9)
	≥ 200, < 500	193,810 (53.1)	21,659 (60.6)	47,004 (52.6)	62,312 (52.1)	62,835 (52.2)
	≥ 500	151,346 (41.5)	11,690 (32.7)	37,018 (41.4)	50,950 (42.6)	51,688 (42.9)
**Admission type**	Elective	318,430 (87.2)	31,166 (87.3)	77,079 (86.3)	103,694 (86.7)	106,491 (88.4)
	Emergency	28,871 (7.9)	2851 (8.0)	6743 (7.5)	9706 (8.1)	9571 (7.9)
	NA	17,824 (4.9)	1695 (4.7)	5539 (6.2)	6219 (5.2)	4371 (3.6)
**Charlson Comorbidity Index**	0–1	15,492 (4.2)	1140 (3.2)	4343 (4.9)	5473 (4.6)	4536 (3.8)
	2–3	278,015 (76.1)	27,289 (76.4)	67,029 (75.0)	90,317 (75.5)	93,380 (77.5)
	4–5	40,879 (11.2)	3722 (10.4)	9942 (11.1)	13,730 (11.5)	13,485 (11.2)
	≥ 6	30,739 (8.4)	3561 (10.0)	8047 (9.0)	10,099 (8.4)	9032 (7.5)
**Barthel Index**	100	316,490 (86.7)	31,333 (87.7)	77,639 (86.9)	103,298 (86.4)	104,220 (86.5)
	65–95	19,040 (5.2)	1606 (4.5)	4504 (5.0)	6357 (5.3)	6573 (5.5)
	45–60	7443 (2.0)	655 (1.8)	1719 (1.9)	2454 (2.1)	2615 (2.2)
	5–40	5919 (1.6)	532 (1.5)	1464 (1.6)	2015 (1.7)	1908 (1.6)
	0	6162 (1.7)	503 (1.4)	1525 (1.7)	2087 (1.7)	2047 (1.7)
	NA	10,071 (2.8)	1083 (3.0)	2510 (2.8)	3408 (2.8)	3070 (2.5)
**Smoking history**	Yes	149,686 (41.0)	13,689 (38.3)	34,799 (38.9)	49,255 (41.2)	51,943 (43.1)
	No	185,061 (50.7)	19,164 (53.7)	47,020 (52.6)	60,322 (50.4)	58,555 (48.6)
	NA	30,378 (8.3)	2859 (8.0)	7542 (8.4)	10,042 (8.4)	9935 (8.2)
**Malnutrition**^b^	Yes	71,374 (19.5)	6789 (19.0)	17,020 (19.0)	23,130 (19.3)	24,435 (20.3)
	No	293,751 (80.5)	28,923 (81.0)	72,341 (81.0)	96,489 (80.7)	95,998 (79.7)
**Level of food intake independence**	Require no assistance	343,025 (93.9)	33,720 (94.4)	83,998 (94.0)	112,146 (93.8)	113,161 (94.0)
	Require partial assistance	10,472 (2.9)	919 (2.6)	2520 (2.8)	3394 (2.8)	3639 (3.0)
	Require full assistance	7838 (2.1)	694 (1.9)	1923 (2.2)	2683 (2.2)	2538 (2.1)
	NA	3790 (1.0)	379 (1.1)	920 (1.0)	1396 (1.2)	1095 (0.9)
**Preoperative oral management**^c,d,e^	Yes	103,231 (28.3)	-	16,527 (18.5)	38,148 (31.9)	48,556 (40.3)
**Preoperative artificial nutrition**^c^	Enteral nutrition^f^	7780 (2.1)	187 (0.5)	1003 (1.1)	3502 (2.9)	3088 (2.6)
	Parenteral nutrition^g^	87,251 (23.9)	10,349 (29.0)	22,996 (25.7)	27,907 (23.3)	25,999 (21.6)
**TNM cancer classification**	I	75,429 (20.7)	8812 (24.7)	20,157 (22.6)	23,804 (19.9)	22,656 (18.8)
	II	79,238 (21.7)	7475 (20.9)	18,784 (21.0)	26,154 (21.9)	26,825 (22.3)
	III	79,374 (21.7)	8078 (22.6)	20,303 (22.7)	25,976 (21.7)	25,017 (20.8)
	IV	30,369 (8.3)	3867 (10.8)	8396 (9.4)	9607 (8.0)	8499 (7.1)
	NA	100,715 (27.6)	7480 (20.9)	21,721 (24.3)	34,078 (28.5)	37,436 (31.1)
**Preoperative cancer treatment**^h^	Chemotherapy	25,231 (6.9)	1737 (4.9)	4786 (5.4)	7460 (6.2)	11,248 (9.3)
	Radiation therapy	4490 (1.2)	384 (1.1)	1019 (1.1)	1543 (1.3)	1544 (1.3)

*BMI* Body mass index, *NA* Not available

^a^Time periods based on year of hospital admission

^b^Defined as BMI < 18.5 for those < 70 years old and BMI < 20 for those ≥ 70 years old

^c^From day of hospital admission to day before surgery

^d^Data only available for 2014 and later

^e^Support for oral intake functions, including swallowing and chewing

^f^Tube feedings prescribed

^g^Intravenous solutions containing amino acids and lipid prescribed

^h^From 60 days before surgery to day before surgery

Among the patient cohort, the surgery sites included the esophagus in 14,784 patients (4.0%), stomach in 103,339 (28.3%), colon in 118,157 (32.4%), rectum in 75,892 (20.8%), liver in 19,277 (5.3%), gallbladder/bile duct in 8,279 (2.3%), and pancreas in 20,568 (5.6%). A total of 190,111 (52.1%) patients underwent laparoscopic surgery (Table 2). The number of patients in each study group was as follows: Period I (2011–2013), 35,712; II (2014–2016), 89,361; III (2017–2019), 119,619; IV (2020–2022), 120,433 (Fig. 1).

**Table 2:** Surgical characteristics of patients who underwent gastrointestinal cancer surgery in the four time periods evaluated^a^

**Characteristics**	**Categories**	**Total**	**Period I** **2011–2013**	**Period II** **2014–2016**	**Period III** **2017–2019**	**Period IV** **2020–2022**
		N = 365125	N = 35712	N = 89361	N = 119619	N = 120433
		n (%)	n (%)	n (%)	n (%)	n (%)
**Surgical site**	Esophagus	14784 (4.0)	1235 (3.5)	3116 (3.5)	4764 (4.0)	5669 (4.7)
	Stomach	103339 (28.3)	12018 (33.7)	28119 (31.5)	33050 (27.6)	30152 (25.0)
	Colon	118157 (32.4)	10641 (29.8)	28040 (31.4)	39330 (32.9)	40146 (33.3)
	Rectum	75892 (20.8)	7320 (20.5)	18096 (20.3)	24622 (20.6)	25854 (21.5)
	Liver	19277 (5.3)	1905 (5.3)	4737 (5.3)	6473 (5.4)	6162 (5.1)
	Gallbladder/bile duct,	8279 (2.3)	712 (2.0)	1867 (2.1)	2854 (2.4)	2846 (2.4)
	Pancreas	20568 (5.6)	1463 (4.1)	4093 (4.6)	6877 (5.7)	8135 (6.8)
	Multiple organs^b^	4829 (1.3)	418 (1.2)	1293 (1.4)	1649 (1.4)	1469 (1.2)
**Surgical method**	Laparoscopic	190111 (52.1)	11941 (33.4)	39354 (44.0)	63725 (53.3)	75091 (62.4)
	Non-laparoscopic	175014 (47.9)	23771 (66.6)	50007 (56.0)	55894 (46.7)	45342 (37.6)
**Medical treatment on day of surgery**	Crystalloid fluid, *mL*					
	≤5,000	92372 (25.3)	7537 (21.1)	22690 (25.4)	30333 (25.4)	31812 (26.4)
	>5,000, ≤10,000	215057 (58.9)	21938 (61.4)	52764 (59.0)	69795 (58.3)	70560 (58.6)
	>10,000	57696 (15.8)	6237 (17.5)	13907 (15.6)	19491 (16.3)	18061 (15.0)
	Colloid fluid, *mL*					
	0	179465 (49.2)	16827 (47.1)	40623 (45.5)	57147 (47.8)	64868 (53.9)
	>0, ≤500	105583 (28.9)	10920 (30.6)	26746 (29.9)	34838 (29.1)	33079 (27.5)
	>500	80077 (21.9)	7965 (22.3)	21992 (24.6)	27634 (23.1)	22486 (18.7)
	Albumin, *mL*					
	0	328915 (90.1)	31778 (89.0)	80039 (89.6)	108079 (90.4)	109019 (90.5)
	>0, ≤500	27871 (7.6)	3093 (8.7)	7272 (8.1)	8765 (7.3)	8741 (7.3)
	>500	8339 (2.3)	841 (2.4)	2050 (2.3)	2775 (2.3)	2673 (2.2)
	Transfusion^c^, *mL*					
	0	320369 (87.7)	30751 (86.1)	77595 (86.8)	104931 (87.7)	107092 (88.9)
	>0, ≤500	17583 (4.8)	1634 (4.6)	4315 (4.8)	5922 (5.0)	5712 (4.7)
	>500	27173 (7.4)	3327 (9.3)	7451 (8.3)	8766 (7.3)	7629 (6.3)
	Intensive care unit admission	152151 (41.7)	11334 (31.7)	32588 (36.5)	51472 (43.0)	56757 (47.1)

^a^Time periods based on year of hospital admission

^b^Surgery involving multiple organs on a single day

^c^Red blood cells, platelets, and/or fresh frozen plasma

### Postoperative feeding route

**Fig. 2:**
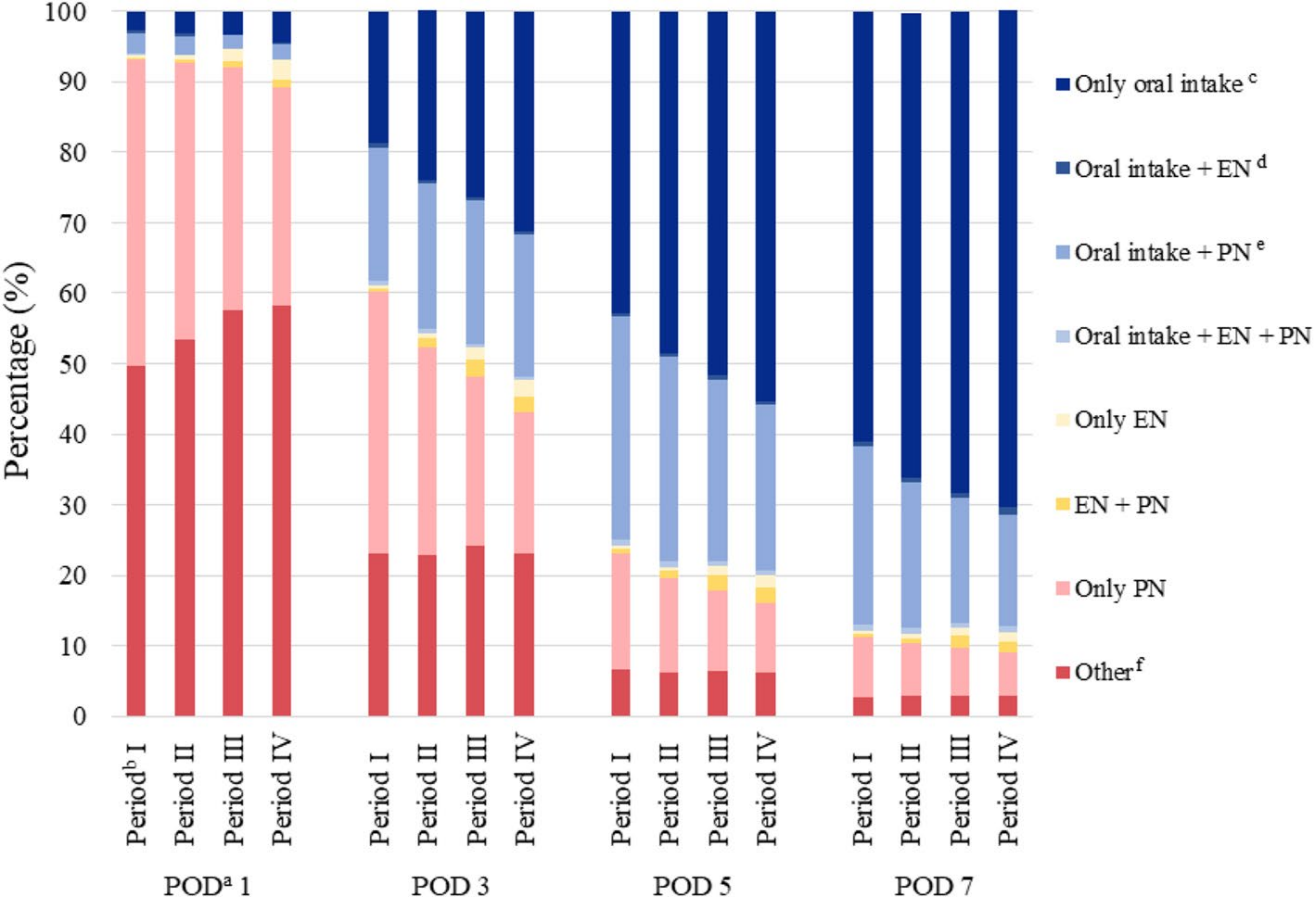
Proportion of feeding routes in patients who underwent gastrointestinal cancer surgery in the four time periods evaluated. The evaluation points were postoperative days 1, 3, 5, and 7. ^a ^Postoperative day (POD) 1 is defined as the next day of the surgery day. ^b^ Time periods based on year of hospital admission: Period I, 2011–2013 (n = 35,712); II, 2014–2016 (n = 89,361); III, 2017–2019 (n = 119,619); IV, 2020–2022 (n = 120,433). ^c^ Oral intake defined as meal served. ^d ^Enteral nutrition (EN) defined as tube feedings prescribed. ^e^ Parenteral nutrition (PN) defined as intravenous solutions containing amino acids and lipid prescribed. ^f^ Other defined as intravenous solutions containing only glucose and electrolytes prescribed

The proportion of feeding routes on POD 1, 3, 5, and 7 is shown in Fig. 2 and Additional file 2. The proportion of patients administered oral intake alone on POD 3 increased significantly from Period I to IV (Period I, 18.7%; II, 24.0%; III, 26.5%; IV, 31.3%; Trend P < 0.001). The proportion of patients who received only EN on POD 3 also increased significantly from Period I to IV (Period I, 0.4%; II, 0.7%; III, 1.8%; IV, 2.4%; P < 0.001). On the other hand, the proportion of patients who received only PN on POD 3 decreased significantly from Period I to IV (Period I, 37.1%; II, 29.5%; III, 24.0%; IV, 19.9%; Trend P < 0.001).

**Fig. 3:**
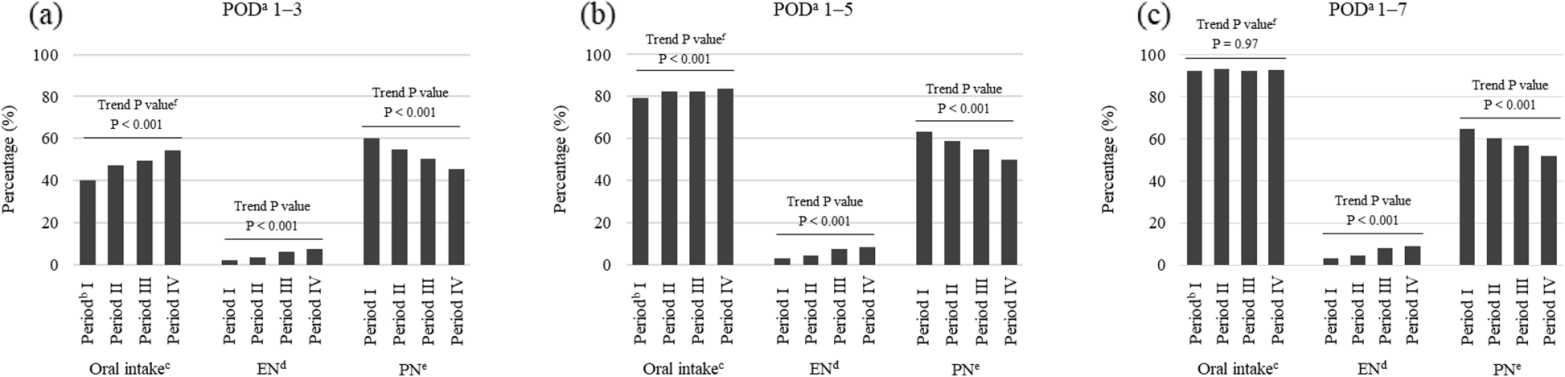
Proportion of feeding routes in patients who underwent gastrointestinal cancer surgery in the four time periods evaluated. The evaluation periods were (a) POD 1 to 3, (b) POD 1 to 5, and (c) POD 1 to 7. ^a^ Postoperative day (POD) 1 is defined as the next day of the surgery day. ^b^ Time periods based on year of hospital admission: Period I, 2011–2013 (n = 35,712); II, 2014–2016 (n = 89,361); III, 2017–2019 (n = 119,619); IV, 2020–2022 (n = 120,433). ^c^ Oral intake defined as meal served. ^d^ Enteral nutrition (EN) defined as tube feedings prescribed. ^e^ Parenteral nutrition (PN) defined as intravenous solutions containing amino acids and lipid prescribed. ^f^ Cochran-Armitage test for trends between groups

The proportion of feeding routes during the period of POD 1 to 3, POD 1 to 5, and POD 1 to 7 is shown in Fig. 3 and Additional file 3. The proportion of patients receiving any oral intake during POD 1 to 3 increased significantly from Period I to IV (Period I, 40.3%; II, 47.1%; III, 49.4%; IV, 54.2%; Trend P < 0.001). The proportion of patients received any EN during POD 1 to 3 also increased significantly from Period I to IV (Period I, 2.3%; II, 3.6%; III, 6.2%; IV, 7.6%; Trend P < 0.001). On the other hand, the proportion of patients received any PN during POD 1 to 3 decreased significantly from Period I to IV (Period I, 60.1%; II, 55.0%; III, 50.3%; IV, 45.5%; Trend P < 0.001).

### PN doses prescribed postoperatively and fulfilment of nutritional goals

**Fig. 4:**
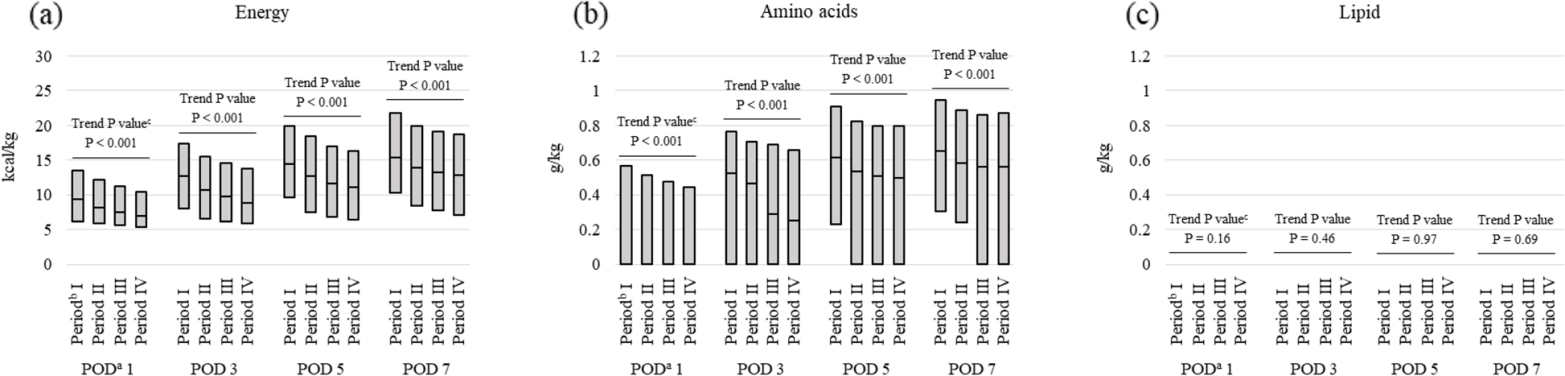
Parenteral doses prescribed in patients who underwent gastrointestinal cancer surgery in the four time periods evaluated. A total of 19,661 patients who fasted from POD 1 to 7 were evaluated. The evaluation points were postoperative days 1, 3, 5, and 7. (a) Energy, (b) amino acid, and (c) lipid doses. Boxplots show medians and interquartile ranges. ^a^ Postoperative day (POD) 1 is defined as the next day of the surgery day. ^b^ Time periods based on year of hospital admission: Period I, 2011–2013 (n = 2507); II, 2014–2016 (n = 5267); III, 2017–2019 (n = 6383); IV, 2020–2022 (n = 5504). ^c^ Jonckheere-Terpstra test for trends between groups

The prescribed parenteral energy, amino acid, and lipid doses on POD 1, 3, 5, and 7 for the 19,661 patients who were fasting from POD 1 to 7 are shown in Fig. 4 and Additional file 4. The median (Q1, Q3) prescribed energy dose (kcal/kg) on POD 7 decreased significantly from Period I to IV [Period I, 15.3 (10.3, 21.9); II, 13.9 (8.4, 20.0); III, 13.2 (7.7, 19.2); IV, 12.9 (7.0, 18.7); Trend P < 0.001]. The median (Q1, Q3) prescribed amino acid dose (g/kg) on POD 7 also decreased significantly from Period I to IV [Period I, 0.65 (0.30, 0.94); II, 0.58 (0.24, 0.89); III, 0.56 (0.00, 0.86); IV, 0.56 (0.00, 0.87); Trend P < 0.001]. The median (Q1, Q3) prescribed lipid dose on POD 7 was 0.00 (0.00, 0.00) g/kg in all study groups (Trend P = 0.69).

**Fig. 5:**
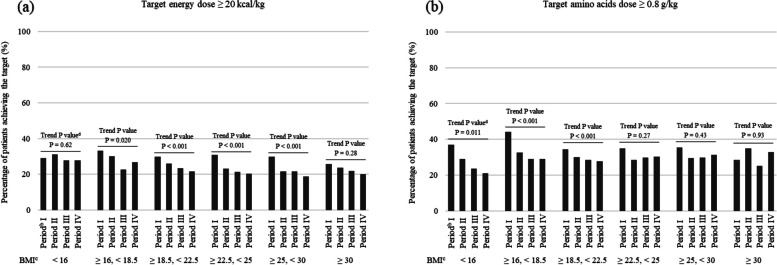
Percentage of patients achieving targets for prescribed doses in the four time periods evaluated. A total of 19,661 patients who fasted from POD 1 to 7 were evaluated. The evaluation point was postoperative day^a^ 7. (a) Energy and (b) amino acid doses. Data are shown by BMI classification. ^a^ Postoperative day (POD) 1 is defined as the next day of the surgery day. ^b^ Time periods based on year of hospital admission: Period I, 2011–2013 (n = 2507); II, 2014–2016 (n = 5267); III, 2017–2019 (n = 6383); IV, 2020–2022 (n = 5504). ^c^ Body mass index (BMI) classification: < 16 (n = 513); ≥ 16, < 18.5 (n = 1990); ≥ 18.5, < 22.5 (n = 7659); ≥ 22.5, < 25 (n = 4951); ≥ 25, < 30 (n = 3914); ≥ 30 (n = 634). ^d^ Cochran-Armitage test for trends between groups

The proportion of patients in each BMI category who were prescribed the target energy or amino acid doses on POD 7 is shown in Fig. 5 and Additional file 5. The proportion of patients who were prescribed the energy goal in the BMI categories ≥ 16 to < 18.5, ≥ 18.5 to < 22.5, ≥ 22.5 to < 25, and ≥ 25 to < 30 decreased significantly from Period I to IV (Trend P < 0.05 for all). The proportion of patients who were prescribed the amino acid goal in the BMI categories < 16, ≥ 16 to < 18.5, and ≥ 18.5 to < 22.5 also decreased significantly from Period I to IV (Trend P < 0.05 for all). Overall, the energy and amino acid doses prescribed in PN did not meet the targets in the majority of patients across the four time periods evaluated.

## Discussion

This study used a nationwide medical claims database to assess changes in the nutritional management of 365,125 patients during the 7 days after GI cancer surgery over a 12-year period (2011–2022). As a result of assessing four 3-year period groups, an increasing proportion of patients were found to receive early oral intake after GI cancer surgery, while a decreasing proportion of patients received early PN. In addition, for patients who fasted from POD 1 to 7 and received PN alone, the prescribed doses of energy and amino acids on POD 7 were found to decrease significantly over the same time period. The energy and amino acid doses prescribed in PN were insufficient in the majority of patients in all four time periods evaluated, and almost no lipid was prescribed in any of the time periods across the patient groups.

A possible explanation for the increasing number of patients receiving early postoperative oral intake from 2011 to 2022 in Japan is the increasing incorporation of ERAS, oral management, and minimally-invasive surgery into clinical practice in Japan. The use of ERAS programs were originally proposed around 2000 and initially implemented in Nordic countries, expanding in Japan since 2010 [14]. Recovery from postoperative GI dysfunction is an important goal within ERAS programs, and early oral intake after surgery is considered one of the most effective ways to achieve this [5]. In Japan, clinicians have become increasingly aware of the importance of early oral intake after surgery as a critical component of ERAS, having been shown to enhance the recovery of GI function, maintain immune function, and reduce complications [7]. The increasing adoption of ERAS programs in Japan may have contributed to the increasing number of patients over time receiving early postoperative oral intake in our study.

The maintenance of oral functions is essential to promote postoperative oral intake. Since 2014, clinicians in Japan have been able to submit medical treatment claims for oral management [24]. Among the study cohort, claims for oral management were submitted for 19% of patients in Period II, increasing markedly to 40% of patients in Period IV. Although the objective of perioperative oral management is the prevention of postoperative aspiration pneumonia and infectious diseases [27, 28], the oral care and feeding-swallowing training included in oral management have also been shown to promote postoperative oral intake [28]. Thus, the increasing use of oral management in Japan may have also contributed to the increasing number of patients over time receiving early postoperative oral intake in our study.

Similar to ERAS and oral management, minimally-invasive surgery in Japan was found to be increasingly used from 2011 to 2022. In fact, the proportion of patients who underwent laparoscopic surgery increased from 33% in Period I to 62% in Period IV. Compared to traditional open surgery, minimally-invasive surgery is known to result in less postoperative pain and fewer complications [10]. In addition, this approach promotes a quicker recovery of GI function, enabling an earlier postoperative oral intake [29]. Thus, the increased use of minimally-invasive GI cancer surgery in Japan may have contributed to the increasing number of patients receiving early postoperative oral intake over the time period evaluated in this study.

In this study, fewer patients were administered PN during the 7 days after GI cancer surgery over time. This may have been the result of more patients receiving early oral intake. However, another reason for this reduced prescribing of PN may be due to the concerns of clinicians regarding complications caused by PN overfeeding. Overfeeding is the provision of energy to critically ill patients in excess of metabolic requirements. In the EPaNIC Trial, a large-scale randomized study conducted in 2011 involving patients in the ICU at risk of malnutrition, patients who had early initiation of PN (i.e., within 48 h after ICU admission) experienced longer ICU stays and more complications than those who had late initiation (i.e., on or after 8th day of ICU admission) [30]. The authors speculated that some of the poorer outcomes associated with early PN overfeeding during the invasive period may have been the result of the suppression of autophagy, with inadequate clearance of cell damage and microorganisms. Other studies have also reported a potential association between overfeeding and increased inflammatory reactions, as well as decreased immune function [31, 32]. Prescribed parenteral energy and amino acids decreased over time in our study (Fig. 4). We assume potential two reasons as follows. First, the EPaNIC Trial suggested the potential adverse effects of overfeeding by PN. This may cause to refrain from administering PN. Second, the understanding of ERAS has encouraged clinicians to start oral intake earlier over time (Fig. 2), and, in turn, discouraged them to provide sufficient PN.

Contrary to the findings of the EPaNIC Trial, other studies have reported on the benefits of early PN administration [33, 34]. This has led to a lack of clear recommendations regarding the optimal timing for the initiation of PN. For example, the Japanese Guidelines for Nutritional Therapy in Critically Ill Patients do not provide clear recommendations on the appropriate time to initiate PN in patients who are unable to receive sufficient nutrition using EN [35]. Thus, an increasing awareness of oral intake and the potential complications associated with early PN overfeeding during critical illness most likely decreased use of PN within 7 days after GI cancer surgery, as observed in our study.

In the subgroup of patients who were fasting from POD 1 to 7 and receiving PN, the proportion of patients achieving their target goals for prescribed parenteral energy and amino acid doses on POD 7 were approximately 20–30% and 20–35%, respectively. The energy and amino acid doses prescribed in PN were also found to be insufficient in the majority of patients in all four time periods. Furthermore, that almost no lipid was prescribed in any of the time periods. Measures for promoting oral intake, such as ERAS and oral management, are most often directed by a multidisciplinary team. Conversely, for patients who have had GI cancer surgery and are fasting, the implementation of PN is often the responsibility of surgeons. However, surgeons may not always have enough time for nutritional management, since their work is primarily focused on performing surgery and general perioperative management [36]. In addition, a study of questionnaire survey about nutritional support in 357 surgeons in the UK showed that 69% of responders replied lack of knowledge and 56% of responders replied lack of time as the barrier for implementation of nutritional management by surgeons [37]. Another study of questionnaire survey in 224 surgeons in Japan showed that approximately 20% or more of responders did not clearly answer the postoperative doses of protein/amino acids [38]. Most surgeons may not have an awareness and full understanding of optimal nutritional management. This may explain why most patients in our study were prescribed doses of parenteral energy and amino acids below guideline recommendations, and no lipids.

A potential solution could involve surgeons proactively seeking the involvement of registered dietitians in the nutritional management of patients after GI cancer surgery. Since 2022 in Japan, medical treatment claims can be submitted and a fee paid when perioperative nutritional management is conducted in acute care hospitals by surgeons and registered dietitians in collaboration or when nutritional management is conducted by registered dietitians working in the patient wards of Special Functioning Hospitals [39]. The proactive involvement of registered dietitians in the nutritional management of patients in the surgical ward could play an important role in facilitating nutritional management and helping patients to achieve their nutritional goals. An increased involvement of registered dietitians in the care of patients undergoing GI cancer surgery may help address inadequate PN doses, and may ultimately contribute to improving the postoperative outcomes of this patient population.

### Limitations

The medical claims database used in this study may be prone to input errors, missing data, and inaccuracies. As this was not a database ideally suited for a clinical study, some information on nutritional management was unavailable. For example, no data was available regarding the reasons for the selection of specific feeding routes. As a result, we were unable to assess whether appropriate feeding routes had been selected. In addition, we were unable to survey the amounts of energy, amino acids, and lipid actually received by patients during oral intake or EN. Furthermore, because information regarding the amounts of PN discarded was lacking, the energy, amino acid, and lipid doses actually administered to fasting patients may have been lower than the prescribed doses used in the study. Nonetheless, our study highlighted the knowledge-to-action gap in clinical practices and is useful for future prospective studies.

## Conclusions

Over the period of 2011 to 2022, an increasing number of patients were found to be administered early oral intake after GI cancer surgery in Japan, compared to a decrease in the number of patients receiving early PN. During the same period, fasting patients who were prescribed PN were administered energy and amino acid doses far below the guideline recommendations, as well as almost no lipids. The results highlight the fact that patients who have recently undergone GI cancer surgery may benefit from a more active promotion of the early use of postoperative oral intake. The careful consideration of PN management is warranted to facilitate achieving the nutritional goals of fasting patients post-GI cancer surgery in Japan.

## Supplementary Information

Additional file 1: Medical claims codes (PDF)

Additional file 2: Proportions of feeding routes in patients who underwent gastrointestinal cancer surgery in the four time periods evaluated (PDF)

Additional file 3: Proportions of feeding routes in patients who underwent gastrointestinal cancer surgery in the four time periods evaluated (PDF)

Additional file 4: Prescribed parenteral doses in patients who underwent gastrointestinal cancer surgery in the four time periods evaluated (PDF)

Additional file 5: Percentages of patients achieving targets for prescribed doses in patients in the four time periods evaluated (PDF)

## Data Availability

If requested, the authors will provide the data or will cooperate fully in obtaining and providing the data on which the manuscript is based for examination by the editors or their assignees.

## Abbreviations

BMI: Body mass index
EN: Enteral nutrition
ERAS: Enhanced recovery after surgery
GI: Gastrointestinal
ICD: International Statistical Classification of Diseases
ICU: Intensive care unit
PN: Parenteral nutrition
POD: Postoperative day
Q1: First quartile
Q3: Third quartile
SD: Standard deviation
SPN: Supplemental parenteral nutrition
TNM: Tumor-Node-Metastasis
UMIN: University Hospital Medical Information Network
WHO: World Health Organization
