# Supplementary material for: Changes in nutritional management after gastrointestinal cancer surgery over a 12-year period: a cohort study using a nationwide medical claims database
Source: BMC Nutr. 2025 Jan 22;11:19. doi: 10.1186/s40795-025-01006-4 (PMC11753049; doi:10.1186/s40795-025-01006-4)
Supplement: Supplementary file 1 — Additional file 1: Medical claims codes (PDF) [file 40795_2025_1006_MOESM1_ESM.pdf]

Table 1. Demographic and clinical characteristics of patients who underwent gastrointestinal cancer surgery in the four time periods evaluated[a].

**Characteristics**	***Categories***	**Total**	**Period I 2011–2013**	**Period II 2014–2016**	**Period III 2017–2019**	**Period IV 2020–2022**
		N = 365125	N = 35712	N = 89361	N = 119619	N = 120433
		n (%)	n (%)	n (%)	n (%)	n (%)
**Age**, *years*	18–59	50951 (14.0)	5914 (16.6)	12966 (14.5)	15964 (13.3)	16107 (13.4)
	60–69	95481 (26.2)	10916 (30.6)	26482 (29.6)	31807 (26.6)	26276 (21.8)
	70–79	138844 (38.0)	12409 (34.7)	32086 (35.9)	45299 (37.9)	49050 (40.7)
	80–89	73296 (20.1)	6048 (16.9)	16519 (18.5)	24326 (20.3)	26403 (21.9)
	≥90	6553 (1.8)	425 (1.2)	1308 (1.5)	2223 (1.9)	2597 (2.2)
**Sex**	Male	229281 (62.8)	22639 (63.4)	56306 (63.0)	75488 (63.1)	74848 (62.1)
	Female	135844 (37.2)	13073 (36.6)	33055 (37.0)	44131 (36.9)	45585 (37.9)
**BMI**	<16	7842 (2.1)	748 (2.1)	1863 (2.1)	2564 (2.1)	2667 (2.2)
	≥16, <18.5	36369 (10.0)	3647 (10.2)	8882 (9.9)	11714 (9.8)	12126 (10.1)
	≥18.5, <22.5	145649 (39.9)	14786 (41.4)	36221 (40.5)	47295 (39.5)	47347 (39.3)
	≥22.5, <25	93240 (25.5)	9301 (26.0)	22937 (25.7)	30776 (25.7)	30226 (25.1)
	≥25, 30	70679 (19.4)	6318 (17.7)	16962 (19.0)	23540 (19.7)	23859 (19.8)
	≥30	11346 (3.1)	912 (2.6)	2496 (2.8)	3730 (3.1)	4208 (3.5)
**Beds in admission hospital**	<200	19969 (5.5)	2363 (6.6)	5339 (6.0)	6357 (5.3)	5910 (4.9)
	≥200, <500	193810 (53.1)	21659 (60.6)	47004 (52.6)	62312 (52.1)	62835 (52.2)
	≥500	151346 (41.5)	11690 (32.7)	37018 (41.4)	50950 (42.6)	51688 (42.9)

**Admission type**	Elective	318430 (87.2)	31166 (87.3)	77079 (86.3)	103694 (86.7)	106491 (88.4)
	Emergency	28871 (7.9)	2851 (8.0)	6743 (7.5)	9706 (8.1)	9571 (7.9)
	NA	17824 (4.9)	1695 (4.7)	5539 (6.2)	6219 (5.2)	4371 (3.6)
**Charlson Comorbidity Index**	0–1	15492 (4.2)	1140 (3.2)	4343 (4.9)	5473 (4.6)	4536 (3.8)
	2–3	278015 (76.1)	27289 (76.4)	67029 (75.0)	90317 (75.5)	93380 (77.5)
	4–5	40879 (11.2)	3722 (10.4)	9942 (11.1)	13730 (11.5)	13485 (11.2)
	≥6	30739 (8.4)	3561 (10.0)	8047 (9.0)	10099 (8.4)	9032 (7.5)
**Barthel Index**	100	316490 (86.7)	31333 (87.7)	77639 (86.9)	103298 (86.4)	104220 (86.5)
	65–95	19040 (5.2)	1606 (4.5)	4504 (5.0)	6357 (5.3)	6573 (5.5)
	45–60	7443 (2.0)	655 (1.8)	1719 (1.9)	2454 (2.1)	2615 (2.2)
	5–40	5919 (1.6)	532 (1.5)	1464 (1.6)	2015 (1.7)	1908 (1.6)
	0	6162 (1.7)	503 (1.4)	1525 (1.7)	2087 (1.7)	2047 (1.7)
	NA	10071 (2.8)	1083 (3.0)	2510 (2.8)	3408 (2.8)	3070 (2.5)
**Smoking history**	Yes	149686 (41.0)	13689 (38.3)	34799 (38.9)	49255 (41.2)	51943 (43.1)
	No	185061 (50.7)	19164 (53.7)	47020 (52.6)	60322 (50.4)	58555 (48.6)
	NA	30378 (8.3)	2859 (8.0)	7542 (8.4)	10042 (8.4)	9935 (8.2)
**Malnutrition**[b]	Yes	71374 (19.5)	6789 (19.0)	17020 (19.0)	23130 (19.3)	24435 (20.3)
	No	293751 (80.5)	28923 (81.0)	72341 (81.0)	96489 (80.7)	95998 (79.7)
**Level of food intake independence**	Require no assistance	343025 (93.9)	33720 (94.4)	83998 (94.0)	112146 (93.8)	113161 (94.0)
	Require partial assistance	10472 (2.9)	919 (2.6)	2520 (2.8)	3394 (2.8)	3639 (3.0)
	Require full assistance	7838 (2.1)	694 (1.9)	1923 (2.2)	2683 (2.2)	2538 (2.1)
	NA	3790 (1.0)	379 (1.1)	920 (1.0)	1396 (1.2)	1095 (0.9)

**Preoperative oral management**[c,d,e]	Yes	103231 (28.3)	-	16527 (18.5)	38148 (31.9)	48556 (40.3)
**Preoperative artificial nutrition**[c]	Enteral nutrition[f]	7780 (2.1)	187 (0.5)	1003 (1.1)	3502 (2.9)	3088 (2.6)
	Parenteral nutrition[g]	87251 (23.9)	10349 (29.0)	22996 (25.7)	27907 (23.3)	25999 (21.6)
**TNM cancer classification**	I	75429 (20.7)	8812 (24.7)	20157 (22.6)	23804 (19.9)	22656 (18.8)
	II	79238 (21.7)	7475 (20.9)	18784 (21.0)	26154 (21.9)	26825 (22.3)
	III	79374 (21.7)	8078 (22.6)	20303 (22.7)	25976 (21.7)	25017 (20.8)
	IV	30369 (8.3)	3867 (10.8)	8396 (9.4)	9607 (8.0)	8499 (7.1)
	NA	100715 (27.6)	7480 (20.9)	21721 (24.3)	34078 (28.5)	37436 (31.1)
**Preoperative cancer treatment**[h]	Chemotherapy	25231 (6.9)	1737 (4.9)	4786 (5.4)	7460 (6.2)	11248 (9.3)
	Radiation therapy	4490 (1.2)	384 (1.1)	1019 (1.1)	1543 (1.3)	1544 (1.3)

[a] Time periods based on year of hospital admission.

[b] Defined as BMI <18.5 for those < 70 years old and BMI <20 for those ≥70 years old.

[c] From day of hospital admission to day before surgery.

[d] Data only available for 2014 and later.

[e] Support for oral intake functions, including swallowing and chewing.

[f] Tube feedings prescribed.

[g] Intravenous solutions containing amino acids and lipid prescribed.

[h] From 60 days before surgery to day before surgery.

BMI, body mass index; NA, not available.
