# Supplementary material for: Changes in nutritional management after gastrointestinal cancer surgery over a 12-year period: a cohort study using a nationwide medical claims database
Source: BMC Nutr. 2025 Jan 22;11:19. doi: 10.1186/s40795-025-01006-4 (PMC11753049; doi:10.1186/s40795-025-01006-4)
Supplement: Supplementary file 2 — Additional file 2: Proportions of feeding routes in patients who underwent gastrointestinal cancer surgery in the four time periods evaluated (PDF) [file 40795_2025_1006_MOESM2_ESM.pdf]

Table 2. Surgical characteristics of patients who underwent gastrointestinal cancer surgery in the four time periods evaluated[a].

Characteristics	*Categories*	Total<br>N = 365125	Period I<br>2011–2013<br>N = 35712	Period II<br>2014–2016<br>N = 89361	Period III<br>2017–2019<br>N = 119619	Period IV<br>2020–2022<br>N = 120433
		n (%)	n (%)	n (%)	n (%)	n (%)
**Surgical site**	Esophagus	14784 (4.0)	1235 (3.5)	3116 (3.5)	4764 (4.0)	5669 (4.7)
	Stomach	103339 (28.3)	12018 (33.7)	28119 (31.5)	33050 (27.6)	30152 (25.0)
	Colon	118157 (32.4)	10641 (29.8)	28040 (31.4)	39330 (32.9)	40146 (33.3)
	Rectum	75892 (20.8)	7320 (20.5)	18096 (20.3)	24622 (20.6)	25854 (21.5)
	Liver	19277 (5.3)	1905 (5.3)	4737 (5.3)	6473 (5.4)	6162 (5.1)
	Gallbladder/bile duct,	8279 (2.3)	712 (2.0)	1867 (2.1)	2854 (2.4)	2846 (2.4)
	Pancreas	20568 (5.6)	1463 (4.1)	4093 (4.6)	6877 (5.7)	8135 (6.8)
	Multiple organs[b]	4829 (1.3)	418 (1.2)	1293 (1.4)	1649 (1.4)	1469 (1.2)
**Surgical method**	Laparoscopic	190111 (52.1)	11941 (33.4)	39354 (44.0)	63725 (53.3)	75091 (62.4)
	Non-laparoscopic	175014 (47.9)	23771 (66.6)	50007 (56.0)	55894 (46.7)	45342 (37.6)
**Medical treatment on day of surgery**	Crystalloid fluid, *mL*					
	≤ 5000	92372 (25.3)	7537 (21.1)	22690 (25.4)	30333 (25.4)	31812 (26.4)
	> 5000, ≤ 10000	215057 (58.9)	21938 (61.4)	52764 (59.0)	69795 (58.3)	70560 (58.6)
	> 10000	57696 (15.8)	6237 (17.5)	13907 (15.6)	19491 (16.3)	18061 (15.0)
	Colloid fluid, *mL*					
	0	179465 (49.2)	16827 (47.1)	40623 (45.5)	57147 (47.8)	64868 (53.9)
	> 0, ≤ 500	105583 (28.9)	10920 (30.6)	26746 (29.9)	34838 (29.1)	33079 (27.5)
	> 500	80077 (21.9)	7965 (22.3)	21992 (24.6)	27634 (23.1)	22486 (18.7)
	Albumin, *mL*					
	0	328915 (90.1)	31778 (89.0)	80039 (89.6)	108079 (90.4)	109019 (90.5)
	> 0, ≤ 500	27871 (7.6)	3093 (8.7)	7272 (8.1)	8765 (7.3)	8741 (7.3)

> 500	8339 (2.3)	841 (2.4)	2050 (2.3)	2775 (2.3)	2673 (2.2)
Transfusion[c], *mL*					
0	320369 (87.7)	30751 (86.1)	77595 (86.8)	104931 (87.7)	107092 (88.9)
> 0, ≤ 500	17583 (4.8)	1634 (4.6)	4315 (4.8)	5922 (5.0)	5712 (4.7)
> 500	27173 (7.4)	3327 (9.3)	7451 (8.3)	8766 (7.3)	7629 (6.3)
Intensive care unit admission	152151 (41.7)	11334 (31.7)	32588 (36.5)	51472 (43.0)	56757 (47.1)

[a] Time periods based on year of hospital admission.

[b] Surgery involving multiple organs on a single day.

[c] Red blood cells, platelets, and/or fresh frozen plasma.
