# Supplementary material for: Changes in nutritional management after gastrointestinal cancer surgery over a 12-year period: a cohort study using a nationwide medical claims database
Source: BMC Nutr. 2025 Jan 22;11:19. doi: 10.1186/s40795-025-01006-4 (PMC11753049; doi:10.1186/s40795-025-01006-4)
Supplement: Supplementary file 3 — Additional file 3: Proportions of feeding routes in patients who underwent gastrointestinal cancer surgery in the four time periods evaluated (PDF) [file 40795_2025_1006_MOESM3_ESM.pdf]

Additional file 3. Proportions of feeding routes in patients who underwent gastrointestinal cancer surgery in the four time periods evaluated[a]. The evaluation periods were postoperative days 1 to 3, 1 to 5, and 1 to 7.

Day[b] range	*Feeding route*[c]	**Period I** **2011–2013** N = 35712	**Period II** **2014–2016** N = 89361	**Period III** **2017–2019** N = 119619	**Period IV** **2020–2022** N = 120433	**Trend P value**[d]
		n (%)	n (%)	n (%)	n (%)	
**POD 1–3**	Oral intake	14399 (40.3)	42047 (47.1)	59065 (49.4)	65231 (54.2)	< **0.001**
	EN	836 (2.3)	3195 (3.6)	7407 (6.2)	9130 (7.6)	< **0.001**
	PN	21474 (60.1)	49140 (55.0)	60119 (50.3)	54817 (45.5)	< **0.001**
**POD 1–5**	Oral intake	28256 (79.1)	73728 (82.5)	98387 (82.3)	101051 (83.9)	< **0.001**
	EN	1043 (2.9)	3816 (4.3)	8859 (7.4)	10326 (8.6)	< **0.001**
	PN	22615 (63.3)	52487 (58.7)	65407 (54.7)	60132 (49.9)	< **0.001**
**POD 1–7**	Oral intake	32977 (92.3)	83100 (93.0)	110245 (92.2)	111692 (92.7)	0.97
	EN	1149 (3.2)	4139 (4.6)	9442 (7.9)	10884 (9.0)	< **0.001**
	PN	23119 (64.7)	53873 (60.3)	67539 (56.5)	62395 (51.8)	< **0.001**

[a] Time periods based on year of hospital admission.

[b] Postoperative day (POD) 1 is defined as the next day of the surgery day.

[c] Oral intake defined as meals served, Enteral Nutrition (EN) as tube feedings prescribed, Parenteral Nutrition (PN) as intravenous solutions containing amino acids and lipid prescribed.

[d] Cochran-Armitage test for trends between groups.
