# Supplementary material for: Changes in nutritional management after gastrointestinal cancer surgery over a 12-year period: a cohort study using a nationwide medical claims database
Source: BMC Nutr. 2025 Jan 22;11:19. doi: 10.1186/s40795-025-01006-4 (PMC11753049; doi:10.1186/s40795-025-01006-4)
Supplement: Supplementary file 4 — Additional file 4: Prescribed parenteral doses in patients who underwent gastrointestinal cancer surgery in the four time periods evaluated (PDF) [file 40795_2025_1006_MOESM4_ESM.pdf]

Additional file 4. Prescribed parenteral doses in patients who underwent gastrointestinal cancer surgery in the four time periods evaluated[a]. A number of 19,661 patients who fasted from postoperative days 1 to 7 were evaluated. The evaluation points were postoperative days 1, 3, 5, and 7.

**Parenteral doses**	***Day***[b]	**Period I** **2011–2013** N = 2507	**Period II** **2014–2016** N = 5267	**Period III** **2017–2019** N = 6383	**Period IV** **2020–2022** N = 5504	**Trend P value**[c]
**Energy**, *kcal/kg* median (Q1, Q3)	POD 1	9.3 (6.2, 13.5)	8.2 (5.9, 12.2)	7.5 (5.7, 11.3)	6.9 (5.4, 10.4)	< **0.001**
	POD 3	12.8 (8.0, 17.4)	10.7 (6.5, 15.6)	9.8 (6.1, 14.6)	8.8 (5.9, 13.8)	< **0.001**
	POD 5	14.5 (9.7, 19.9)	12.8 (7.4, 18.4)	11.7 (6.8, 17.0)	11.1 (6.4, 16.3)	< **0.001**
	POD 7	15.3 (10.3, 21.9)	13.9 (8.4, 20.0)	13.2 (7.7, 19.2)	12.9 (7.0, 18.7)	< **0.001**
**Amino acids**, *g/kg* median (Q1, Q3)	POD 1	0.00 (0.00, 0.57)	0.00 (0.00, 0.51)	0.00 (0.00, 0.48)	0.00 (0.00, 0.45)	< **0.001**
	POD 3	0.53 (0.00, 0.77)	0.47 (0.00, 0.71)	0.29 (0.00, 0.69)	0.25 (0.00, 0.66)	< **0.001**
	POD 5	0.61 (0.23, 0.91)	0.53 (0.00, 0.82)	0.51 (0.00, 0.80)	0.50 (0.00, 0.80)	< **0.001**
	POD 7	0.65 (0.30, 0.94)	0.58 (0.24, 0.89)	0.56 (0.00, 0.86)	0.56 (0.00, 0.87)	< **0.001**
**Lipid**, *g/kg* median (Q1, Q3)	POD 1	0.00 (0.00, 0.00)	0.00 (0.00, 0.00)	0.00 (0.00, 0.00)	0.00 (0.00, 0.00)	0.16
	POD 3	0.00 (0.00, 0.00)	0.00 (0.00, 0.00)	0.00 (0.00, 0.00)	0.00 (0.00, 0.00)	0.46
	POD 5	0.00 (0.00, 0.00)	0.00 (0.00, 0.00)	0.00 (0.00, 0.00)	0.00 (0.00, 0.00)	0.97
	POD 7	0.00 (0.00, 0.00)	0.00 (0.00, 0.00)	0.00 (0.00, 0.00)	0.00 (0.00, 0.00)	0.69
**Energy**, *kcal/kg* mean (SD)	POD 1	10.4 (8.8)	9.3 (5.0)	8.8 (4.7)	8.1 (4.4)	< **0.001**
	POD 3	13.5 (7.1)	11.9 (6.8)	11.1 (6.5)	10.4 (6.2)	< **0.001**
	POD 5	15.6 (8.7)	13.8 (7.9)	12.9 (7.6)	12.4 (7.4)	< **0.001**
	POD 7	16.6 (8.4)	15.1 (8.4)	14.3 (8.3)	13.9 (8.0)	< **0.001**

**Amino acids**, *g/kg* mean (SD)	POD 1	0.31 (0.40)	0.27 (0.36)	0.23 (0.35)	0.21 (0.34)	< **0.001**
	POD 3	0.49 (0.40)	0.42 (0.39)	0.38 (0.40)	0.36 (0.41)	< **0.001**
	POD 5	0.57 (0.43)	0.52 (0.41)	0.48 (0.41)	0.47 (0.41)	< **0.001**
	POD 7	0.61 (0.40)	0.56 (0.40)	0.54 (0.41)	0.53 (0.41)	< **0.001**
**Lipid**, *g/kg* mean (SD)	POD 1	0.02 (0.08)	0.02 (0.09)	0.02 (0.09)	0.02 (0.08)	0.16
	POD 3	0.04 (0.14)	0.03 (0.11)	0.03 (0.12)	0.03 (0.11)	0.46
	POD 5	0.05 (0.15)	0.05 (0.14)	0.05 (0.14)	0.04 (0.14)	0.97
	POD 7	0.06 (0.17)	0.06 (0.16)	0.06 (0.16)	0.06 (0.15)	0.69

[a] Time periods based on year of hospital admission.

[b] Postoperative day (POD) 1 is defined as the next day of the surgery day.

[c] Jonckheere-Terpstra test for trends between groups.

Q1, quartile 1; Q3, quartile 3; SD, standard deviation
