# Supplementary material for: Changes in nutritional management after gastrointestinal cancer surgery over a 12-year period: a cohort study using a nationwide medical claims database
Source: BMC Nutr. 2025 Jan 22;11:19. doi: 10.1186/s40795-025-01006-4 (PMC11753049; doi:10.1186/s40795-025-01006-4)
Supplement: Supplementary file 5 — Additional file 5: Percentages of patients achieving targets for prescribed doses in patients in the four time periods evaluated (PDF) [file 40795_2025_1006_MOESM5_ESM.pdf]

Additional file 5. Percentages of patients achieving targets for prescribed doses in patients in the four time periods evaluated[a]. A number of 19,661 patients fasted from postoperative days[b] 1 to 7 were evaluated. The evaluation point was postoperative day 7. Data was shown by BMI classification.

Target dose	Body Mass Index (BMI) classification[c]	Period I 2011–2013 N = 2507	Period II 2014–2016 N = 5267	Period III 2017–2019 N = 6383	Period IV 2020–2022 N = 5504	Trend P value[d]
		n/N (%)	n/N (%)	n/N (%)	n/N (%)	
**Energy** *Target dose ≥20 kcal/kg*	<16	19/65 (29.2)	43/138 (31.2)	49/177 (27.7)	37/133 (27.8)	0.62
	≥16, <18.5	80/242 (33.1)	168/559 (30.1)	139/612 (22.7)	155/577 (26.9)	**0.02**
	≥18.5, <22.5	310/1042 (29.8)	537/2070 (25.9)	577/2452 (23.5)	451/2095 (21.5)	**< 0.001**
	≥22.5, <25	192/623 (30.8)	309/1335 (23.1)	343/1607 (21.3)	281/1386 (20.3)	**< 0.001**
	≥25, <30	138/461 (29.9)	221/1022 (21.6)	283/1316 (21.5)	209/1115 (18.7)	**< 0.001**
	≥30	19/74 (25.7)	34/143 (23.8)	48/219 (21.9)	40/198 (20.2)	0.28
**Amino acids** *Target dose ≥0.8 g/kg*	<16	24/65 (36.9)	40/138 (29.0)	42/177 (23.7)	28/133 (21.1)	**0.01**
	≥16, <18.5	107/242 (44.2)	182/559 (32.6)	178/612 (29.1)	168/577 (29.1)	**< 0.001**
	≥18.5, <22.5	360/1042 (34.5)	620/2070 (30.0)	698/2452 (28.5)	583/2095 (27.8)	**< 0.001**
	≥22.5, <25	217/623 (34.8)	380/1335 (28.5)	478/1607 (29.7)	418/1386 (30.2)	0.27
	≥25, <30	163/461 (35.4)	302/1022 (29.5)	392/1316 (29.8)	350/1115 (31.4)	0.43
	≥30	21/74 (28.4)	50/143 (35.0)	55/219 (25.1)	65/198 (32.8)	0.93

[a] Time periods based on year of hospital admission.

[b] Postoperative day 1 is defined as the next day of the surgery day.

[c] BMI classification: <16 (n = 513); ≥16, <18.5 (n = 1990); ≥18.5, <22.5 (n = 7659); ≥22.5, <25 (n = 4951); ≥25, <30 (n = 3914); ≥30 (n = 634).

[d] Cochran-Armitage test for trends between groups.
